# External Validation of the JAKPOT Score for Diagnosing *JAK2*-Positive Erythrocytosis: A Retrospective Cohort Study †

**DOI:** 10.3390/jcm14155173

**Published:** 2025-07-22

**Authors:** Justin Bruni Senecal, Yasmine Madan, Rabia Tahir, Sabina Rajkumar, Wendy Lim, Mark Crowther, Siraj Mithoowani

**Affiliations:** 1 Department of Medicine, McMaster University, Hamilton, ON L8S 4L8, Canada; justin.senecal@medportal.ca (J.B.S.); rabia.tahir@medportal.ca (R.T.); limwp@mcmaster.ca (W.L.); crowthrm@mcmaster.ca (M.C.); 2 Health Sciences Program, McMaster University, Hamilton, ON L8S 4L8, Canada; madany@mcmaster.ca (Y.M.); rajkus11@mcmaster.ca (S.R.)

**Keywords:** erythrocytosis, Janus Kinase 2, polycythemia vera, erythropoietin

## Abstract

**Background/Objectives**: Erythrocytosis is a common laboratory abnormality affecting approximately 4% of males and 0.4% of females. The JAKPOT score was recently developed to differentiate primary from secondary erythrocytosis without molecular testing. JAKPOT+ patients meet any of the following criteria: erythrocytes > 6.45 × 1012/L, platelets > 350 × 109/L, or neutrophils > 6.2 × 109/L. We aimed to validate this score and identify predictors of JAK2-positive erythrocytosis in a retrospective cohort. **Methods**: We identified 213 patients (50 female, mean age 57 years) with undifferentiated erythrocytosis, serum erythropoietin (EPO) and JAK2 molecular testing (V617F or exon 12) at a tertiary care center in Hamilton, Canada, between 2017 and 2022. Charts were manually reviewed for laboratory data, comorbidities, demographics, and medications. We evaluated the diagnostic accuracy of EPO, JAKPOT, and a combination of low EPO and JAKPOT (EPO-JAKPOT) for predicting JAK2 mutant erythrocytosis. Multivariate logistic regression analysis was performed to detect predictors of JAK2 mutant erythrocytosis. **Results**: Forty patients (19%) had JAK2 mutations. Older age (*p* < 0.01), higher platelet count (*p* < 0.01), and lower EPO (*p* < 0.01) were associated with JAK2 mutant erythrocytosis in a multivariate analysis. JAKPOT+ status had a sensitivity of 0.88 (95% CI, 0.73–0.94). Combining low EPO or JAKPOT+ status into a new score (EPO-JAKPOT) increased sensitivity to 0.95 (95% CI, 0.83–0.98). Restricting JAK2 testing to only EPO-JAKPOT+ patients would have led to 55% fewer molecular tests in our cohort. **Conclusions**: The EPO-JAKPOT score shows promise in excluding JAK2 mutant erythrocytosis without molecular testing, but further prospective validation is warranted.

## 1. Introduction

Erythrocytosis, defined by the World Health Organization (WHO) as a hemoglobin (Hb) greater than 165 g/L for males or 160 g/L for females [1], is a common laboratory finding affecting up to 4% of males and 0.4% of females [2]. The large majority of such individuals have secondary erythrocytosis, whereas polycythemia vera (PV)—the most common cause of primary erythrocytosis—is relatively rare, with an incidence of only 0.84 cases per 100,000 individuals [3,4].

The hallmark laboratory criteria for diagnosing PV are an elevated Hb and/or hematocrit (Hct), low serum erythropoietin (EPO), mutated Janus Kinase 2 (*JAK2*) and a bone marrow biopsy showing hypercellularity for age, trilineage growth (panmyelosis) and pleomorphic, mature megakaryocytes [5]. In isolation, low serum EPO lacks sensitivity for PV, such that 80% of patients with PV have a normal EPO level [6]. Activating mutations in *JAK2* are present in 98% of patients with polycythemia vera [7], but molecular genetic testing is relatively costly and is not universally available across clinical laboratories. Therefore, there is an unmet need for a reliable, cost-effective, and widely accessible strategy to differentiate between PV from secondary erythrocytosis.

Chin-Yee et al. (2023) [8] recently developed the JAKPOT score as a screening test for *JAK2* mutant erythrocytosis. The JAKPOT prediction rule includes only three parameters from the complete blood count (CBC) in patients who have not been treated for suspected PV: (i) absolute red blood cells > 6.45 × 10^12^/L, (ii) platelets > 350 × 10^9^/L, and (iii) neutrophils > 6.2 × 10^9^/L. In the JAKPOT derivation study, the absence of all three criteria ruled out *JAK2* mutant erythrocytosis with a sensitivity of 95–100% and a false negative rate of 0.4%, but the score has not been externally validated.

Our aim was to externally validate the JAKPOT score in a retrospective cohort of patients investigated for erythrocytosis at a tertiary care hospital. Our secondary aims were to identify predictors of *JAK2* mutant erythrocytosis in our cohort.

## 2. Methods and Materials

### 2.1. Study Population

We conducted a retrospective cohort study at a 777-bed tertiary care hospital in Hamilton, Canada. Adult (>18 years of age) inpatients and outpatients with both an EPO level and *JAK2* molecular testing performed between 31 December 2017 and 31 December 2022 were included. Patients were excluded if they were anemic (hemoglobin < 115 g/L) at the time of EPO testing, had incomplete records or were known to have donated blood or phlebotomized in the three months before testing. Recently phlebotomized patients were excluded because phlebotomy would confound the interpretation of the hemoglobin, hematocrit and EPO level. This study was approved by the Hamilton Integrated Research Ethics Board (#15535).

### 2.2. Data Extraction and Laboratory Assays

We reviewed electronic medical records for demographic data, comorbidities, laboratory parameters, imaging studies, molecular test results for *JAK2* V617F or exon 12 mutations, pathology results, pulmonary function test results, and medications, including anticoagulants, antiplatelets, diuretics, testosterone, and sodium–glucose transporter-2 (SGLT2) inhibitors. We recorded laboratory parameters from the same day that *JAK2* molecular testing was ordered; if unavailable, we recorded the most recent values obtained before molecular testing. We recorded information from clinical notes as close as possible to the date of EPO testing in a RedCAP database [9]. Data were extracted by JS, YM, RT, and SR. Eighty charts (convenience sample) were extracted in duplicate to ensure consistency between reviewers. Complete blood counts (CBC) were performed on the Sysmex XN-3000 analyzer. EPO testing was performed by enzyme linked immunosorbent assay (Bio-Techne, Minneapolis, MN, USA, R&D Systems). *JAK2* molecular testing was performed by an allele specific polymerase chain reaction (AS-PCR) followed by allele separation and detection using the 4200 TapeStation System (Agilent Technologies, Santa Clara, CA, USA) with a lower limit of detection of 1–5% mutant allele burden.

### 2.3. Outcomes

Our primary objective was to determine the sensitivity (Sn), specificity (Sp), positive likelihood ratio (+LR), and negative likelihood ratio (−LR) of serum EPO, JAKPOT, and a combination of EPO and JAKPOT to identify patients with *JAK2* mutant erythrocytosis. Our secondary objective was to identify adjusted and unadjusted clinical and laboratory predictors of *JAK2* mutant erythrocytosis.

### 2.4. Statistical Analysis

We summarized patient characteristics descriptively with categorical variables expressed as percentages and quantitative variables as means with standard deviations. We calculated the Sn, Sp, +LR, and −LR of low EPO, JAKPOT, and EPO-JAKPOT for the diagnosis of *JAK2* mutant erythrocytosis from 2 × 2 confusion matrices and high EPO for JAK2 mutant negative erythrocytosis (Supplementary Materials, Tables S1–S4). Low EPO was defined as <3.8 mU/mL, which is the lower limit of the reference interval for our institution’s assay. Sn was defined as True Positives [TP]/(TP + False Negatives [FN]), and Sp was defined as True Negatives [TN]/(TN + False Positives [FP]). A total of 95% confidence intervals (CI) were calculated using the Wilson efficient score method [10].

We identified univariate clinical and laboratory predictors of *JAK2* mutant erythrocytosis with Mann–Whitney U-tests, two-tailed Welch *t*-tests, Chi-squared tests, and Fisher exact tests where appropriate and performed a multivariate logistic regression analysis with a subset of the predictors. Predictors in the multivariate analysis were chosen based on the completeness of the data and on theoretical grounds rather than by automated variable selection. Multicollinearity was assessed by calculation of variance inflation factors (Supplementary Materials, Table S5). Twenty-nine (14%) missing ferritin values in the logistic regression analysis were imputed using multiple imputation by chained equations [11]. A sensitivity analysis was performed showing results of the logistic regression analysis without missing value imputation (Supplementary Materials, Table S6). *p* < 0.05 was used to denote statistical significance for all analyses. *p* values were corrected using the Holm–Bonferroni method for multiple comparisons. We used Python (v3.11.9) for statistical analyses with packages numpy (v1.26.4), pandas (v2.2.2), statsmodels (v0.14.2), pingouin (v0.5.3), scipy (v1.13.1), and scikit-learn (v1.4.2).

## 3. Results

**Figure 1:**
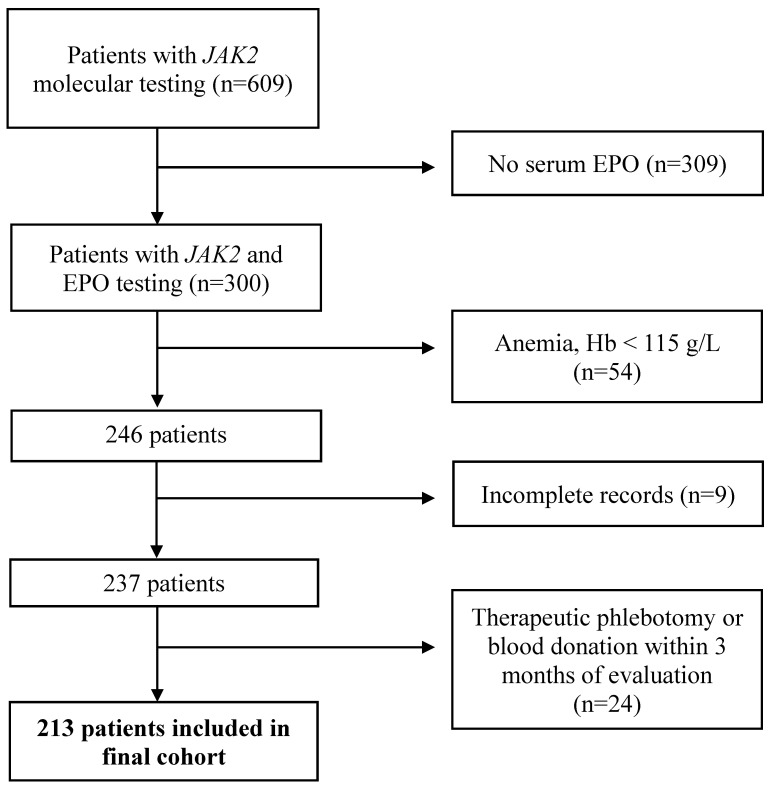
Cohort identification. EPO: serum erythropoietin, Hb: hemoglobin.

We identified 609 patients with *JAK2* testing performed between 31 December 2017 and 31 December 2022. We included 213 patients after excluding patients who were anemic, had no serum EPO measured, had incomplete records, or were known to have undergone therapeutic phlebotomy or who donated blood within three months of testing (Figure 1). The mean age of included patients was 57.7 years, and 163 patients were male (69%) (Table 1). The mean hemoglobin was 174 g/L, mean serum EPO level was 10.7 mU/mL, and 40 (19%) patients had a *JAK2* mutation (39 [18%] *JAK2* V617F, and 1 patient with a *JAK2* exon 12 mutation). Ten patients (5%) were on sodium–glucose cotransporter 2 (SGLT2) inhibitors; all were JAK2-negative (Table 1). No patients were receiving cytoreductive therapy before testing.

### 3.1. Performance of Low EPO or JAKPOT to Diagnose JAK2-Positive Erythrocytosis

**Figure 2:**
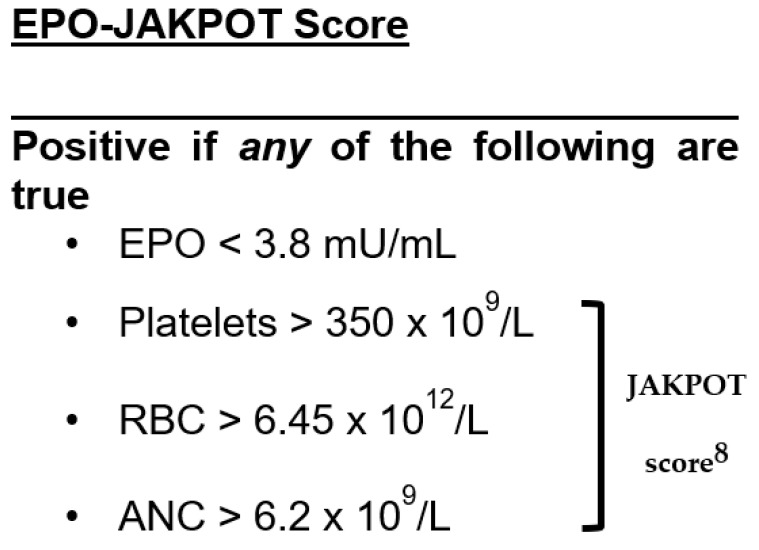
EPO-JAKPOT score. EPO: serum erythropoietin, RBC: absolute red blood cell count, ANC: absolute neutrophil count.

Low EPO (<3.8 mU/mL) had a Sn of 0.77 (95% CI, 0.62–0.87), Sp of 0.98 (95% CI, 0.94–0.99), −LR of 0.23 (95% CI, 0.13–0.41), and +LR of 33 (95% CI, 12.5–89.6) for the diagnosis of *JAK2* mutant erythrocytosis (Table 2, and Supplementary Materials Table S1). Elevated EPO (>16.9 mU/mL) had a Sn of 0.16 (95% CI, 0.11–0.22), Sp of 1.0 (95% CI, 0.91–1.0), −LR of 0.84, and an infinite +LR for the diagnosis of *JAK2* negative (secondary) erythrocytosis (Table S4). A positive JAKPOT score (Figure 2) had a Sn of 0.88 (95% CI, 0.73–0.94), Sp of 0.65 (95% CI, 0.57–0.72), −LR of 0.19 (95% CI, 0.08–0.44), and +LR 2.5 (95% CI, 2.0–3.2) for the diagnosis of *JAK2* mutant erythrocytosis (Table 2, and Supplementary Materials Table S2).

### 3.2. Performance of EPO-JAKPOT to Diagnose JAK2-Positive Erythrocytosis

Patients with a low EPO level (<3.8 U/mL) and/or positive JAKPOT score were classified as EPO-JAKPOT positive (Figure 2). Adding EPO to the JAKPOT score improved sensitivity for diagnosing JAK2 mutant erythrocytosis without sacrificing specificity. A positive EPO-JAKPOT score had a Sn of 0.95 (95% CI, 0.83–0.98), Sp of 0.66 (95% CI, 0.58–0.72), −LR of 0.07 (95% CI, 0.02–0.30), and +LR of 2.7 (95% CI, 2.2–3.4) to diagnose JAK2 mutant erythrocytosis (Table 2, and Supplemental Materials Table S3).

### 3.3. Univariate Predictors of JAK2-Positive Erythrocytosis

Patients with *JAK2*-positive erythrocytosis had a lower mean EPO than patients with *JAK2*-negative erythrocytosis (4.4 mU/mL vs. 12.3 mU/mL; *p* < 0.01) (Table 1). Older age, higher platelet count, higher hematocrit, higher absolute RBC count, and lower ferritin were also associated with *JAK2*-positive erythrocytosis (*p* < 0.01 for all comparisons) (Table 1).

### 3.4. Adjusted (Multivariate) Predictors of JAK2-Positive Erythrocytosis

Older age (*p* < 0.01), lower EPO (<3.8 mU/mL) (*p* < 0.01), and higher platelet count (*p* < 0.01) were independently associated with *JAK2* mutant erythrocytosis after adjusting for potential confounders (Table 3). Looking specifically at components of the JAKPOT score, only the platelet count (*p* < 0.01) was associated with *JAK2* mutant erythrocytosis after adjustment (Table 3).

## 4. Discussion

In our cohort, the JAKPOT score was less sensitive for identifying patients with *JAK2*-positive erythrocytosis compared to the JAKPOT derivation study (Sn 0.88; 95% CI 0.74–0.95 vs. 0.95–1.0) [8]. We hypothesized that combining JAKPOT with low EPO into a unified score (EPO-JAKPOT) would improve diagnostic performance compared to using either JAKPOT or EPO alone. We found that EPO-JAKPOT had high sensitivity (0.95; 95% CI 0.83–0.98) and a low −LR (0.07; 95% CI 0.02–0.30) for *JAK2* mutant erythrocytosis, meaning that primary erythrocytosis could potentially be ruled out in an EPO-JAKPOT negative patient.

We found that low EPO (<3.8 mU/mL) was highly specific (0.97; 95% CI, 0.94–0.99) but insensitive (0.76; 95% CI 0.61–0.86) for diagnosing *JAK2* mutant erythrocytosis. Likewise, high EPO (>16.9 mU/mL) had high specificity (1.0; 95% CI 0.89–1.0) but low sensitivity (0.16; 95% CI, 0.11–0.21) for *JAK2*-negative (secondary) erythrocytosis. In other words, an EPO level outside of the assay’s reference range accurately classified patients as *JAK2* mutant or *JAK2* wild type, but most EPO levels in our cohort (70%) were in the normal range and, therefore, unhelpful. These findings are consistent with other retrospective studies, which report that EPO levels below 2.9 or 3.7 mU/mL are specific (Sp 0.94–0.98) but relatively insensitive (Sn 0.64–0.68) for diagnosing PV [12,13]. In one retrospective cohort, a sensitivity of 0.96 for *JAK2* mutant erythrocytosis was achieved only after increasing the EPO cut-off to 7.15 mU/mL [6]. Likewise, elevated EPO (>15.1 mU/mL) is known to have high specificity for a diagnosis of secondary erythrocytosis (Sp 0.98) but is insensitive (Sn 0.47) [12].

Older age, low EPO, and higher platelet count predicted *JAK2*-positivity in our cohort after adjusting for confounders. Additional univariate predictors included higher hematocrit (*p* < 0.01), and lower ferritin (*p* < 0.01). Low ferritin also approached statistical significance in our multivariate analysis (Table 3). These findings are overall consistent with other studies evaluating clinical and laboratory characteristics of patients with PV [8,13,14] and, when present, may prompt clinicians to consider a diagnosis of primary erythrocytosis.

Ten patients (5%), all of whom were JAK2-negative, were on SGLT2-inhibitors. The clinical significance and optimal management of SGLT-2 inhibitor-induced erythrocytosis remains uncertain [15,16,17].

We speculate that a higher prevalence of *JAK2* positivity (19% compared with 12%) [8] and our exclusion of patients known to have recently undergone therapeutic phlebotomy or blood donation may help explain why the sensitivity of JAKPOT differed in our cohort compared to the derivation study. The two cohorts were similar in terms of severity of erythrocytosis (mean hemoglobin 175 g/L vs. 177 g/L), mean age (57 vs. 58 years) and proportion of male patients (69 vs. 72%) and were both assembled from tertiary care hospitals. Other unreported differences in patient characteristics, including, for example, kidney function, baseline ferritin, or risk factors for secondary erythrocytosis (e.g., proportion of smoking patients), may also have played a role.

The strengths of our study were our inclusion of all patients with EPO and *JAK2* testing at our institution over a 5-year period and our ability to collect detailed laboratory and clinical information on these patients. Limitations include our relatively small sample size, leading to wide confidence intervals, selection bias resulting from patients investigated at a tertiary care hospital, collection of laboratory data at only a single point in time, and incomplete data for some clinical and laboratory parameters (e.g., ferritin and spleen size) due to the retrospective nature of data collection. JAK2 exon 12 mutation testing was ordered at the discretion of the consulting physician and may have led to some missed *JAK2* mutant cases. Similar to the JAKPOT score, our outcome of interest was detecting *JAK2* mutant (primary) erythrocytosis rather than identifying patients with a formal WHO diagnosis of polycythemia vera. Weaknesses of our multivariate analysis include low event rate and multicollinearity between a subset of the predictors, but findings from this analysis are consistent with other cohorts. One drawback of EPO-JAKPOT, compared to the original JAKPOT score, is the need to measure serum EPO. However, EPO testing is recommended for the workup of undifferentiated erythrocytosis [18], and low EPO remains a minor criterion in the WHO 2018 criteria for the diagnosis of polycythemia vera.

Had our study cohort been screened with EPO-JAKPOT, we would have ruled out *JAK2*-positive erythrocytosis in 130 patients (55%) without molecular testing at the cost of only 2 missed cases (1%). The prevalence of *JAK2* mutant erythrocytosis in primary care is unknown, but it is likely less than 1% compared to 19% in our cohort [19]. We expect that EPO-JAKPOT would have even better negative predictive value in primary care and other settings with a lower prevalence of JAK2 mutant erythrocytosis than what was observed in our study [20]. In summary, the EPO-JAKPOT score shows potential for excluding *JAK2* mutant erythrocytosis in select patients without molecular testing. However, additional prospective validation is required before it can be adopted into routine clinical practice.

## Figures and Tables

**Table 1:** Patient characteristics and univariate predictors of *JAK2* mutant erythrocytosis.

	All Patients (*n* = 213)	*JAK2* Positive (*n* = 40)	*JAK2* Negative (*n* = 173)	*p* Value (Nominal)	*p* Value (Corrected *)
Age, years (mean [SD])	57.7 (15.2)	66.1 (11.4)	54 (15.7)	<0.01	<0.01
Male (N [%])	163 (69%)	22 (55%)	123 (71%)	0.08	1.0
BMI, kg/m^2^ (mean [SD])	33.0 (7.9)	28.4 (4.6)	32.9 (8.5)	<0.01	0.09
Weight, kg (mean [SD])	91.4 (19.5)	84.1 (15.1)	93.2 (20.2)	0.02	0.42
**Comorbidities**					
OSA (N [%])	45 (21%)	3 (8%)	42 (24%)	0.02	0.34
COPD (N [%])	18 (8%)	3 (8%)	15 (9%)	1.00	1.0
Current smoking (N [%])	65 (30%)	4 (10%)	61 (35%)	<0.01	0.03
Prior history of smoking (N [%])	52 (23%)	10 (25%)	42 (24%)	1.0	1.0
Any VTE (N [%])	17 (8%)	6 (16%)	11 (6%)	0.10	1.0
Acute coronary syndrome (N [%])	16 (8%)	4 (10%)	12 (7%)	0.51	1.0
Ischemic stroke (N [%])	5 (2%)	2 (5%)	3 (2%)	0.24	1.0
Peripheral arterial disease (N [%])	3 (1%)	1 (3%)	2 (1%)	0.47	1.0
Chronic kidney disease (N [%])	3 (1%)	1 (3%)	2 (1%)	1.00	1.0
Cancer (N [%]) **	1 (0.5%)	1 (3%)	0 (0%)	0.19	1.0
**Medications**					
Any diuretic (N [%])	42 (20%)	4 (10%)	38 (22%)	0.12	1.0
SGLT2 Inhibitor (N [%])	10 (5%)	0 (0%)	10 (6%)	0.21	1.0
Testosterone (N [%])	21 (9%)	1 (2.5%)	20 (12%)	0.14	1.0
Any antiplatelet (N [%])	57 (27%)	14 (35%)	43 (25%)	0.23	1.0
Any anticoagulant (N [%])	23 (11%)	6 (15%)	17 (10%)	0.40	1.0
**Laboratory parameters**					
EPO, mU/mL (mean [SD]), reference interval 3.8–16.9	10.7 (11.1)	4.4 (1.4)	12.3 (12.3)	<0.01	<0.01
Hemoglobin, g/L (mean [SD]), reference interval: 115–180	174 (16.3)	179 (20.1)	172 (14.7)	0.05	0.83
Hematocrit, L/L (mean [SD]), reference interval 0.37–0.54	0.52 (0.05)	0.56 (0.06)	0.51 (0.05)	<0.01	<0.01
Absolute red blood cells, 10^9^ cells/L (mean [SD]), reference interval 3.8–6.5	5.8 (0.74)	6.4 (0.98)	5.7 (0.60)	<0.01	<0.01
Platelets, 10^9^ cells/L (mean [SD]), reference interval 150–400	279.1 (158.1)	461.0 (217.4)	236.9 (103.0)	<0.01	<0.01
Absolute neutrophils, 10^9^ cells/L (mean [SD]), reference interval 2.0–7.5	6.0 (4.5)	8.5 (8.0)	5.5 (2.9)	0.02	0.42
Ferritin, μg/L (mean [SD]), reference interval 30–100	151.3 (181.7)	63.1 (75.9)	170.6 (192.2)	<0.01	<0.01
**Imaging studies**					
Spleen size, cm (mean [SD]) 71 patients	12.0 (2.5)	13.7 (3.0)	11.4 (2.1)	0.01	0.19
Liver size, cm (mean [SD]) 71 patients	16.7 (2.3)	16.1 (2.9)	16.9 (2.2)	0.37	1.0

SD: standard deviation, BMI: body mass index, SGLT2: sodium–glucose cotransporter-2, OSA: obstructive sleep apnea, COPD: chronic obstructive pulmonary disease, VTE: venous thromboembolism, EPO: serum erythropoietin. Due to incomplete data, some characteristics are only known for a subset of patients: BMI (*n* = 95), weight (*n* = 111), ferritin (*n* = 184), spleen size (*n* = 71), liver size (*n* = 71) * Holm–Bonferroni correction for multiple comparisons ** Renal cell carcinoma, hepatocellular carcinoma, or cerebellar hemangioblastoma.

**Table 2:** Performance of serum EPO, JAKPOT, and EPO-JAKPOT for diagnosing *JAK2* mutant erythrocytosis.

	**Number of Patients** **(N [%])**	***JAK2*+** **(N [%])**	**Sensitivity (95% CI)**	**Specificity** **(95% CI)**	**Negative Likelihood Ratio (95% CI)**	**Positive Likelihood Ratio (95% CI)**
**EPO**						
Low (<3.8 mU/mL)	35 (16%)	31 (88%)	0.77 (0.62–0.87)	0.98 (0.94–0.99)	0.23 (0.13–0.41)	33 (12.5–89.6)
Normal (3.8–16.9 mU/mL)	150 (70%)	9 (6%)				
High (>16.9 mU/mL)	28 (13%)	0 (0%)				
JAKPOT *						
Positive	95 (45%)	35 (36%)	0.88 (0.73–0.94)	0.65 (0.57–0.72)	0.19 (0.08–0.44)	2.5 (2.0–3.2)
Negative	118 (55%)	5 (4%)				
EPO-JAKPOT **						
Positive	98 (46%)	38 (39%)	0.95 (0.83–0.98)	0.66 (0.58–0.72)	0.07 (0.02–0.30)	2.7 (2.2–3.4)
Negative	115 (54%)	2 (2%)				

* JAKPOT positive: 1 or more of red blood cells > 6.45 × 10^12^ cells/L, platelets > 350 × 10^9^ cells/L, absolute neutrophils > 6.2 × 10^9^ cells/L; ** EPO-JAKPOT positive: 1 or more of red blood cells > 6.45 × 10^12^ cells/L, platelets > 350 × 10^9^ cells/L, absolute neutrophils > 6.2 × 10^9^ cells/L, EPO < 3.8 mU/mL; EPO: erythropoietin, *JAK2*+: mutant Janus Kinase 2 (V617F or exon 12).

**Table 3:** Adjusted predictors of *JAK2* mutant erythrocytosis by multivariate logistic regression.

	Odds for *JAK2*-Positive Erythrocytosis (95% CI)	*p* Value
Age (years)	1.10 (1.04–1.16)	<0.01
Male sex	1.12 (0.28–4.37)	0.88
EPO (mU/mL)	0.50 (0.32–0.76)	<0.01
ANC (10^9^ cells/L)	1.06 (0.84–1.33)	0.63
RBC (10^9^ cells/L)	1.69 (0.61–4.63)	0.31
Platelets (10^9^ cells/L)	1.01 (1.00–1.01)	<0.01
Ferritin (μg/L) *	0.99 (0.98–1.00)	0.06

* 29 missing ferritin values were imputed using multiple imputation by chained equations (Stef van Buuren, Karin Groothuis-Oudshoorn (2011) [11]; ANC: absolute neutrophil count, RBC: red blood cell count, EPO: serum erythropoietin.

## Data Availability

The datasets generated during and/or analyzed during the current study are not publicly available to preserve patient confidentiality, but a subset of fully anonymized data is available from the corresponding author on reasonable request.

## Supplementary Materials

The following supporting information can be downloaded at: https://www.mdpi.com/article/10.3390/jcm14155173/s1.
