# Supplementary material for: External Validation of the JAKPOT Score for Diagnosing JAK2-Positive Erythrocytosis: A Retrospective Cohort Study
Source: J Clin Med. 2025 Jul 22;14(15):5173. doi: 10.3390/jcm14155173 (PMC12347527; doi:10.3390/jcm14155173)
Supplement: Supplementary file 1 [file jcm-14-05173-s001.zip › jcm-3651068-supplementary.pdf]

Supplemental Materials

Table S1. Confusion matrix for low EPO in predicting *JAK2* mutant erythrocytosis

	**JAK2+**	**JAK2-**
**Low EPO (<3.8 mU/mL)**	TP: 31	FP: 4
**Normal or high EPO (≥ 3.8 mU/mL)**	FN: 9	TN: 169

TP: True Positive, FP: False Positive, FN: False Negative, TN: True Negative, EPO: serum erythropoietin

Table S2. Confusion matrix for JAKPOT in predicting *JAK2* mutant erythrocytosis

	**JAK2+**	**JAK2-**
**JAKPOT+**	TP: 35	FP: 60
**JAKPOT-**	FN: 5	TN: 113

TP: True Positive, FP: False Positive, FN: False Negative, TN: True Negative

Table S3. Confusion matrix for EPO-JAKPOT in predicting *JAK2* mutant erythrocytosis

	**JAK2+**	**JAK2-**
**EPO-JAKPOT+**	TP: 38	FP: 60
**EPO-JAKPOT-**	FN: 2	TN: 113

TP: True Positive, FP: False Positive, FN: False Negative, TN: True Negative

Table S4. Confusion matrix for high EPO in predicting *JAK2* mutant negative erythrocytosis

	**JAK2-**	**JAK2+**
**High EPO (>16.9 mU/mL)**	TP: 28	FP: 0
**Normal or Low EPO (≤ 16.9 mU/mL)**	FN: 145	TN: 40

TP: True Positive, FP: False Positive, FN: False Negative, TN: True Negative, EPO: serum erythropoietin

Table S5. Variance inflation factors for multivariate logistic regression analysis

	**Variance inflation factor**
Age (years)	12.88
Male sex	1.58
EPO (mU/mL)	1.95
ANC (10^9 cells/L)	3.21
RBC (10^9 cells/µL)	17.95
Platelets (10^9 cells/µL)	4.91
Ferritin (µg/L)	1.58

Table S6. Sensitivity analysis of a multivariate logistic regression model without imputation of missing ferritin values (n=184 cases)

	**Odds for *JAK2* positive erythrocytosis (95% CI)**	**P value**
Age (years)	1.10 (1.03-1.17)	**<0.01**
Male sex	0.93 (0.18-4.90)	0.93
EPO (mU/mL)	0.47 (0.27-0.80)	**<0.01**
ANC (10^9 cells/L)	1.21 (0.67-2.20)	0.53
RBC (10^9 cells/L)	2.66 (0.75-9.45)	0.13
Platelets (10^9 cells/L)	1.01 (1.00-1.01)	**<0.01**
Ferritin (µg/L)	0.99 (0.98-1.00)	0.08

ANC: absolute neutrophil count, RBC: red blood cell count, EPO: serum erythropoietin
